# Stattic and metformin inhibit brain tumor initiating cells by reducing STAT3-phosphorylation

**DOI:** 10.18632/oncotarget.14159

**Published:** 2016-12-24

**Authors:** Verena Leidgens, Judith Proske, Lisa Rauer, Sylvia Moeckel, Kathrin Renner, Ulrich Bogdahn, Markus J. Riemenschneider, Martin Proescholdt, Arabel Vollmann-Zwerenz, Peter Hau, Corinna Seliger

**Affiliations:** ^1^ Department of Neurology and Wilhelm Sander-NeuroOncology Unit, University Hospital Regensburg, Regensburg, Germany; ^2^ Department of Internal Medicine III, University Hospital Regensburg, Regensburg, Germany; ^3^ Department of Neuropathology, Regensburg University Hospital, Regensburg, Germany; ^4^ Department of Neurosurgery, University Hospital Regensburg, Regensburg, Germany

**Keywords:** glioma, BTIC, STAT3, Stattic, metformin

## Abstract

Glioblastoma (GBM) is the most common and malignant type of primary brain tumor and associated with a devastating prognosis. Signal transducer and activator of transcription number 3 (STAT3) is an important pathogenic factor in GBM and can be specifically inhibited with Stattic. Metformin inhibits GBM cell proliferation and migration. Evidence from other tumor models suggests that metformin inhibits STAT3, but there is no specific data on brain tumor initiating cells (BTICs).

We explored proliferation and migration of 7 BTICs and their differentiated counterparts (TCs) after treatment with Stattic, metformin or the combination thereof. Invasion was measured *in situ* on organotypic brain slice cultures. Protein expression of phosphorylated and total STAT3, as well as AMPK and mTOR signaling were explored using Western blot. To determine functional relevance of STAT3 inhibition by Stattic and metformin, we performed a stable knock-in of STAT3 in selected BTICs.

Inhibition of STAT3 with Stattic reduced proliferation in all BTICs, but only in 4 out of 7 TCs. Migration and invasion were equally inhibited in BTICs and TCs. Treatment with metformin reduced STAT3-phosphorylation in all investigated BTICs and TCs. Combined treatment with Stattic and metformin led to significant additive effects on BTIC proliferation, but not migration or invasion. No additive effects on TCs could be detected. Stable STAT3 knock-in partly attenuated the effects of Stattic and metformin on BTICs.

In conclusion, metformin was found to inhibit STAT3-phosphorylation in BTICs and TCs. Combined specific and unspecific inhibition of STAT3 might represent a promising new strategy in the treatment of glioblastoma.

## INTRODUCTION

High-grade gliomas, especially glioblastomas (GBM), are highly complex and heterogeneous primary brain tumors, accounting for about 30% of all tumors of the central nervous system [1]. Glioblastomas are nearly uniformly fatal with median overall survival ranging between 14.6 and 26.3 months in patients treated within clinical studies [2, 3]. Brain tumor initiating cells (BTICs) represent cancer stem-like progenitor cells, which are not only implicated in tumor initiation, but also in recurrence and progression [4–6]. BTICs are characterized by self-renewal, clonogenicity, pluripotency, and closely resemble the histopathological phenotype of parental tumors after implantation of these cells into athymic mice [6].

Persistent activation of STAT3 (signal transducer and activator of transcription number 3) has been detected in many cancers [7], including gliomas, and is correlated with poor survival [8]. This was confirmed by correlation of strong expression of STAT3 phosphorylated at Y705 in GBM specimens with a more aggressive phenotype and shorter overall survival [9]. A series of elegant studies has demonstrated an important role of STAT3 in gliomas *in vivo* and *in vitro*. Evidence has emerged, that STAT3 is required by BTICs to maintain their stem-like characteristics [10]. RNA interference of STAT3 sufficiently led to growth arrest, inhibited neurosphere-formation and could induce apoptosis in BTICs [11]. Hence, STAT3 inhibitors have become a major interest in neuro-oncology. The STAT inhibitor Stattic [12] was shown to selectively inhibit STAT3 [13]. However, it is still unclear whether Stattic binds directly to the phosphorylation site at Y705 or if it acts by altering the conformation of the SH2 domain, because it binds to Cys687 on the opposite side of the phosphopeptide binding face [13].

Metformin (1,1-dimethylbiguanide hydrochloride) is a biguanide drug mainly prescribed in the treatment of type 2 diabetes [14]. Metformin has also antineoplastic effects and may reduce the risk of certain cancer types in diabetic patients [15, 16]. Several *in vitro* studies revealed anti-proliferative effects of metformin on cancer stem cells [17], glioma initiating cells [18, 19], and human GBM lines [20]. Known mechanisms of action of metformin are the inhibition of complex I of the respiratory chain [21], resulting in activation of AMPK (adenosine monophosphate-activated protein kinase) and the inhibition of mTOR (mammalian Target Of Rapamycin) [22]. Interestingly, metformin has been shown to reduce STAT3-phosphorylation in a study investigating triple-negative breast cancer [23]. Similar effects could also be shown in two established GBM lines [24].

The primary aim of our study was to better characterize the effects of STAT3 inhibition on primary BTICs and their differentiated counterparts. In addition to specific STAT3 inhibition with Stattic, we investigated whether metformin inhibits STAT3-phosphorylation in BTICs and whether additive effects can be achieved by combining Stattic with the approved and clinically well-tolerated anti-diabetic drug metformin.

## RESULTS

### Characteristics of brain tumor initiating cells

All primary BTICs used here were derived from patients, who had undergone resection of WHO grade IV gliomas at the Neurosurgery Department of the University Hospital Regensburg [19, 25]. Primary cell lines were established and used in low (typically below passage 8) passage numbers to assure maximum resemblance to original tumor cells. O^6^-methylguanine-DNA methyltransferase (MGMT) methylation status varied in-between the lines, while all lines were isocitrate dehydrogenase 1-(IDH1) wild type in culture. BTIC-13 lost its IDH1 R132H mutation under culture conditions (Table 1). Cells were kept as BTICs under serum free conditions, and after withdrawal of growth factors and addition of 10% serum to the cell culture medium as differentiated tumor cells (TCs).

**Table 1:** Patient characteristics

	Histology	WHO Grade	MGMT meth.	IDH1	Age	Gender	OS (months)
**BTIC-7**	Primary GBM	IV	–	wt	52	f	16.3
**BTIC-8**	Primary GBM	IV	–	wt	52	f	4.0
**BTIC-10**	Primary GBM	IV	+	wt	46	m	18.8
**BTIC-11**	Primary GBM	IV	+	wt	55	m	17.5
**BTIC-12**	Gliosarcoma	IV	+	wt	69	m	16.5
**BTIC-13**	Secondary GBM	IV	+	R132H	42	m	8.5
**BTIC-18**	Primary GBM	IV	–	wt	49	m	20.5

Seven different BTICs and their differentiated counterparts were used for *in vitro* assays. All BTICs were derived from WHO grade IV gliomas. While MGMT methylation status differed between lines, IDH mutation was negative for all cells in culture (R132H mutation in BTIC-13 was lost under culture conditions).

### Stattic treatment reduces proliferation and migration of BTICs and TCs

**Figure 1:**
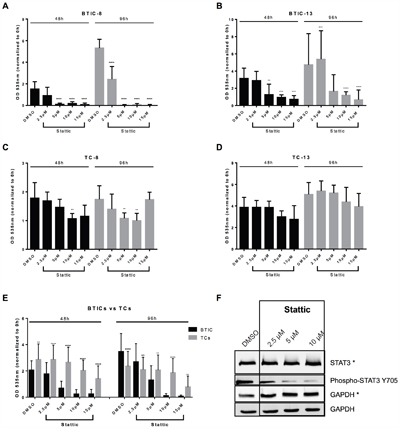
Proliferation is reduced upon Stattic treatment Exemplary proliferation of BTIC-8 **A**. and -13 **B**. and respective TC-8 **C**. and -13 **D**. upon Stattic treatment at indicated concentrations (2.5, 5, 10, 15 μM). **E**. Proliferation of BTICs (summarized for BTIC-7, -8, -10, -11, 12, -13, -18) was affected significantly more than that of the respective TCs (summarized for TC-7, -8, -10, -11, 12, -13, -18). **F**. Stattic treatment (24 h) reduced phosphorylation of STAT3 as exemplarily shown in Western blot analysis of BTIC-11. Corresponding GAPDH controls are indicated by use or not use of the asterisk.

**Figure 2:**
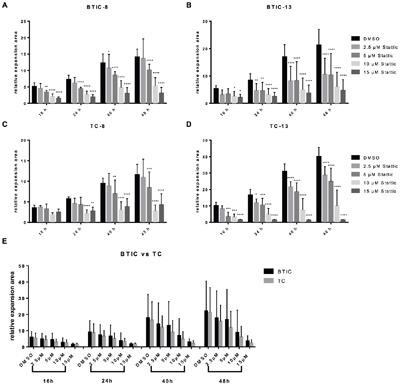
Migration is restricted upon Stattic treatment Exemplary migration of BTIC-8 **A**. and -13 **B**. and respective TC-8 **C**. and -13 **D**. upon Stattic treatment at indicated concentrations (2.5, 5, 10, 15 μM). **E**. Migration of BTICs (summarized for BTIC-8, -11, -13, -18) and respective TCs (summarized for TC-8, -11, -13, -18) was equally affected.

First, we investigated the effects of different doses of Stattic on proliferation (Figure 1) and migration (Figure 2) of BTICs and TCs. Proliferation was assessed after 48 and 96 h to ensure sufficient proliferation while simultaneously avoiding cell death due to high confluence. Migration was analyzed at 16, 24, 40 and 48 h, to provide also early time points (16, 24 h), when migration is not confounded by proliferation. The reduction of proliferation and migration caused by Stattic was dose-dependent and cell line-dependent. High doses of Stattic (10 – 15 μM) inhibited proliferation in all BTIC lines and in 4 out of 7 respective TC lines significantly and in two other lines noticeably (Figure 1 and Supplementary Figure 1). Protein expression of pSTAT3 at Y705 was dose-dependently reduced after Stattic treatment (24 h), while total STAT3 was not affected, as assessed by Western blot (Figure 1F). When investigating migration at early time points (24 h), high doses of Stattic restricted migration in 6 out of 7 BTIC lines and in all TC lines (Figure 2 and Supplementary Figure 2). When comparing sensitivity of BTICs and TCs, BTICs were significantly more sensitive to Stattic than TCs regarding proliferation (Figure 1E), but also differed according to their basal proliferative capacity, which is observable when comparing the DMSO controls. Migration did not differ between BTICs and TCs (Figure 2E).

### STAT3-overexpression enhances proliferation and migration

**Figure 3:**
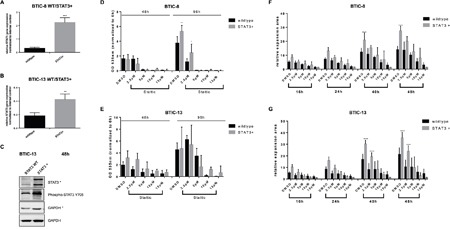
Effects of STAT3-overexpression in BTICs **A, B**. STAT3-overexpression was confirmed by qRT-PCR and Western blot **C**. Corresponding GAPDH controls are indicated by use or not use of the asterisk. **D, E**. Proliferation of wild type and STAT3-overexpressing BTIC-8 and BTIC-13 upon Stattic treatment. **F, G**. Migration of wild type and STAT3-overexpressing BTIC-8 and BTIC-13 upon Stattic treatment.

We assessed effects of STAT3-overexpression on proliferation, migration, and Stattic sensitivity in exemplary cell lines (BTIC-8 and -13). We chose BTIC-8 and -13 due to their good response to Stattic as wild type cells, which unmasks a reduction of inhibitory effects more easily than in cells that already respond less in the wild type state. Markedly increased levels of STAT3 were confirmed via qRT-PCR (Figure 3A, 3B) and Western blot (Figure 3C) upon transfection with the STAT3 construct. Stattic treatment led to weaker effects on STAT3-phosphorylation in STAT3-overexpressing cells (Supplementary Figure 3). Upon STAT3-overexpression, BTIC-8 revealed significantly increased proliferation after 96 h (Figure 3D). Sensitivity to Stattic appeared slightly weaker in both lines for some Stattic concentrations, but the results did not reach statistical significance. Migration was enhanced due to increased STAT3 expression (Figure 3F, 3G), but sensitivity to Stattic did not differ substantially between wild type and knock-in (Figure 3F, 3G).

### BTIC motility is affected by Stattic in an *in situ* 3D model

**Figure 4:**
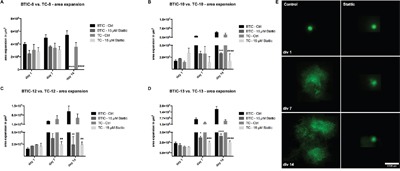
Effects of STAT3 inhibition in BTICs and TCs on OBSCs **A-D**. Spheroid expansion area of BTIC-8, -10, -12 and -13 and respective TCs on OBSCs with or without treatment with 15 μM Stattic every other day. Exemplary pictures of BTIC-13 are shown in (E).

Spheroids of lentivirally transduced and fluorescence tagged BTIC-8, BTIC-10, BTIC-12 and BTIC-13 were implanted on OBSCs (organotypic brain slice cultures). Migration areas were analyzed over 14 days *in vitro* (=div) and normalized to 0-h spheroid expansion areas. Treatment of OBSCs with Stattic (15 μM) led to significantly reduced invasion in all investigated BTICs and TCs (Figure 4A-4D). In BTIC-8 and TC-8 Stattic treatment led to slightly reduced invasion between day 0 and day 7, but the cells were hardly detected anymore at day 14, indicating cytotoxicity. Exemplary pictures of invading BTIC-13 are shown in Figure 4E. BTICs tended to migrate farther than TCs, but both groups had similar migratory potential after treatment with 15 μM Stattic.

### Metformin inhibits STAT3-phosphorylation

**Figure 5:**
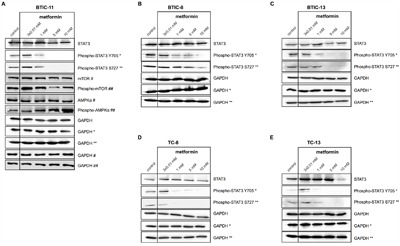
Metformin reduces STAT3-phosphorylation Western blot analyses of BTIC-11 **A**., -8 **B**. and -13 **C**. revealed reduced levels of phosphorylated STAT3 at Y705 and S727 after 48 h of metformin treatment. Activated AMPK and reduced mTOR signaling are displayed as positive controls (A). Similar effects were seen in respective TC-8 **D**. and -13 **E**. Corresponding GAPDH controls are indicated by the same number of asterisks/pounds signs.

Based on the results above and published effects of metformin on BTICs [18, 19], we next investigated STAT3-phosphorylation at Y705 and S727 after treatment with increasing doses of metformin (Figure 5). Following a 48 h treatment, metformin inhibited STAT3-phosphorylation in all investigated BTICs and TCs (Figure 5 and Supplementary Figure 4). Signaling pathways known to be influenced by metformin (activation of AMPK and inhibition of mTOR) are shown as positive control in BTIC-11. STAT3 knock-in opposed (low dose) or weakened (high-dose) the effects of metformin treatment, as shown exemplarily in BTIC-8 (S4D).

### Combined treatment with Stattic and metformin increases functional effects

**Figure 6:**
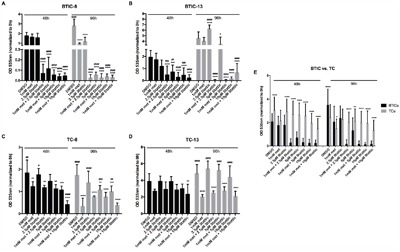
Effects of the combination of Stattic and metformin on proliferation of BTICs and TCs **A, B**. Proliferation of BTIC-8 and -13 and **C, D**. TC-8 and -13 upon treatment with 1 mM metformin without or with the addition of 2.5, 5 and 10 μM Stattic, respectively. **E**. Proliferation of BTICs (summarized for BTIC-7, -8, -10, -11, 12, -13, -18) was affected significantly more by the combination of metformin and Stattic than of respective TCs (summarized for TC-7, -8, -10, -11, 12, -13, -18). Asterisks indicate significant differences as compared to the corresponding DMSO-control, the pound signs indicate significance as compared to 1 mM metformin.

**Figure 7:**
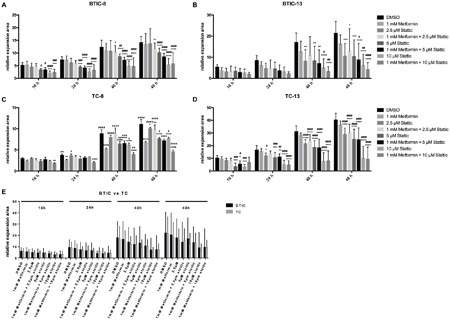
Effects of the combination of Stattic and metformin on migration of BTICs and TCs **A, B**. Migration of BTIC-8 and -13 and **C, D**. TC-8 and -13 upon treatment with 1 mM metformin without or with the addition of 2.5, 5 and 10 μM Stattic, respectively. **E**. Migration of BTICs (summarized for BTIC-7, -8, -10, -11, 12, -13, -18) was not significantly more affected by the combination of metformin and Stattic than that of respective TCs (summarized for TC-7, -8, -10, -11, 12, -13, -18). Asterisks indicate significant differences as compared to the corresponding DMSO-control, the pound signs indicate significance as compared to 1 mM metformin.

Based on the influence of metformin on STAT3-phosphorylation we investigated the effects of combined treatment with Stattic and metformin on proliferation (Figure 6) and migration (Figure 7) of BTICs and TCs. Low dose combination treatment (1 mM metformin and 2.5 μM Stattic) resulted in strong and additive proliferation inhibition in all BTIC lines (Figure 6 and Supplementary Figure 5). In contrast, no additive effects were observed for TC lines (Figure 6 and Supplementary Figure 5). Compared to single treatment, the combination did not result in enhanced restriction of migration as seen in BTICs as well as TCs (Figure 7 and Supplementary Figure 6). In addition, the combination of metformin and Stattic did not lead to additive effects regarding invasion as investigated in BTIC-8 using the aforementioned OBSC model (Supplementary Figure 7).

## DISCUSSION

For the first time, we were able to demonstrate that metformin in combination with specific STAT3 inhibition by Stattic additively reduced proliferation in a multitude of primary human BTICs. Furthermore, we observed that metformin inhibited phosphorylation of STAT3 in all BTICs and TCs. On the contrary, the combination of Stattic and metformin affected migration of BTICs and TCs to a lower extent in all primary lines. Stable knock-in of STAT3 led to a slight attenuation of the observed effects, while proliferation and partly migration was enhanced.

Many studies have been performed to identify and understand aberrant regulation of STAT3 and its impact on proliferation, migration, and invasion. Among those, Bromberg *et al*. [26] reported that dominant-negative STAT3 abrogated oncogenic transformation, whereas constitutively activated STAT3C [27] mutants induced it. Furthermore, STAT3 was shown to be essential in human glioma cells to maintain their tumor initiating capacity and the ability to invade the normal brain [10]. These findings indicated STAT3 as a promising target for anticancer therapy.

Stattic was used in concentrations ranging from 2.5 to 15 μM to reveal minimal dosages required for proliferation and migration restriction *in vitro*. Although *in vitro* studies reported cell cytotoxicity even with low Stattic concentrations, *in vivo* animal studies revealed no signs of severe side effects using Stattic concentrations distinctly exceeding those used *in vitro*. In line with this, Lin et al. [28] demonstrated high cell cytotoxicity rates in GBM cell lines *in vitro* even with low Stattic concentrations (0.73 μM U87 and 0.84 μM U251), whereas others administered 3.75 mg kg^-1^ Stattic [29] or 10 mg kg^-1^ subcutanously [30] without side effects in their animal models. Missing side effects are likely due to the fact that STAT3 may not be essential for viability of normal cells in adult mammalians [7].

According to the cancer stem cell model, tumor recurrence after initial treatment results from remaining, therapy-resistant cancer stem cells (reviewed in [31]). In our study, substantially lower Stattic concentrations were required to restrict proliferation of BTICs than of TCs, indicating a more important role of STAT3 in BTIC proliferation than in TCs or a higher pharmacological susceptibility. Although therapy-resistance of cancer stem cells is widely assumed, it lacks conclusive experimental evidence [31]. Consequently, it may not be excluded that inhibition of a central transcription factor as STAT3 has profound consequences for those tumor cells, namely BTICs, that contain self-renewal and multilineage differentiation capacity in contrast to more differentiated tumor cells, especially since loss of STAT3 was shown to be lethal in embryonic stem cells [32]. STAT3 was shown to be critically involved in cell survival and cell cycle progression, particularly via induction of c-myc and several cyclins [33], as well as to contribute essentially to maintain the ability to invade the normal brain [10]. It is likely that BTICs depend more on STAT3 for their cell cycle progression, while STAT3 contributes equally to migration and proliferation in differentiated tumor cells. In addition, our BTICs were cultured in Epidermal Growth Factor Receptor (EGFR) enriched culture medium. EGFR is commonly upregulated in glioblastomas [34], and high expression of EGFR leads to Akt-activation through the phosphatidylinositol 3-kinase pathway [35] and increased pSTAT3 expression [36]. It therefore seems plausible, that BTICs respond better to STAT3 inhibition due to enhanced expression of the therapeutic target, which will likely also be the case under physiological conditions in the patient.

Stable knock-in of STAT3 slightly attenuated the effects of Stattic on the functional level and on protein expression, which is most likely explained by the fact, that the overexpressing form is constitutively active. The difference of effects of wildtype and STAT3-overexpressing cells however remained only moderate, which might either be explained by insufficient functioning of the constitutively active from of STAT3 or by Stattic not being fully specific for the inhibition of the Tyr705 phosphorylation site.

In line with our observations for Stattic, we observed a similar decrease in STAT3-phosphorylation under metformin treatment. Also, combined treatment with metformin and Stattic led to additive effects and STAT3 knock-in partly abrogated the effects of metformin.

Metformin is an approved and well-tolerated drug for the treatment of type 2 diabetes [37]. In addition, metformin was proven to inhibit proliferation [17, 19, 22, 38, 39] and invasion [40], to induce apoptosis [22, 38, 39] and autophagy [38], and to cause differentiation [18] of glioma cells. The underlying molecular mechanisms include inhibition of mTOR by AMPK-dependent and independent ways and inhibition of Akt signaling [22, 38, 41]. In contrast to Stattic, metformin's action on tumor cells is not limited to STAT3 inhibition. Multi-pathway inhibitory agents, such as metformin, may reduce the risk to induce resistance against therapy and may be more efficient than both specific inhibition of the EGFR/STAT3 and mTOR singaling pathways [42]. Effects of metformin on STAT3 have been described in non-malignant [43–53] and malignant tissues [23, 24, 54–64], with mainly inhibitory effects of metformin on STAT3-phosphorylation [23, 24, 43, 46–64]. However, one study also reported an increased level of STAT3 in the hypothalamus after metformin treatment [45] and two studies did not find a significant effect of metformin on STAT3 in brown adipocytes [44] and astroglial cells [65]. Only one prior study investigated STAT3 after metformin treatment in glioblastoma and observed a reduced phosphorylation at the Y705 binding site, but the results were merely based on two established glioma cell lines [24]. Our study confirmed those first findings in a number of primary BTICs and TCs [23, 24, 54–64], which is closer to the *in vivo* situation.

The exact mechanism, how metformin inhibits phosphorylation of STAT3 has not been fully elucidated yet. Although AMPK has been discussed as a mediator between metformin and STAT3 signaling in prior studies [59], other authors proposed also AMPK-independent effects of metformin on STAT3 signaling [56]. In addition, mTOR was shown to associate with STAT3 and to facilitate STAT3 activation via specific mTOR-dependent phosphorylation at Ser727 [66]. Knowing that metformin inhibits activation of mTOR, this may be an additional mechanism explaining the observed effects and is supported by the fact, that the mTOR inhibitor rapamycin also reduces STAT activation [66]. We did, however, also observe reduced phosphorylation at Y705, which may not be explained by the same, but possibly a similar, yet undescribed, mechanism. For some BTICs and TCs, metformin and Stattic treatment also reduced levels of total STAT3 at high doses. Possibly, functional inactivation of STAT3 by metformin or Stattic may also lead to increased degradation of the total protein with increasing doses.

STAT3 is known to mediate a metabolic switch from oxidative phosphorylation to increased glycolysis [67]. STAT3 inhibition by Stattic therefore reduces STAT3-induced glycolysis and metformin inhibits complex 1 of the respiratory chain. This might represent an additional mechanism, explaining additive effects. Additive effects of pharmacological partners of metformin are highly desirable, as metformin was mostly used in higher doses than usually reached in diabetes treatment in most of the prior studies (among others [17, 38]). The combination of metformin and Stattic therefore allows a reduction of drug doses of the single agents, thereby possibly reducing side effects by maintaining inhibitory effects on tumor cells. However, although metformin was administered at about 10% of the dose used in other studies on glioma cells [17], drug dosing still needs to be intensified in comparison to the usual antidiabetic drug doses to reach concentrations of 1 mM in brain tissue [68].

To date, only a few specific STAT3 inhibitors, but not Stattic, have been translated to clinical trials. For instance, OPB-31121, which specifically inhibits STAT3-phosphorylation was recently investigated in patients with advanced solid tumors (but not brain tumors) in a phase I trial [69] and showed the potential to stabilize (n = 8) or reduce (n = 2) tumors (n = 18).

In summary, combining specific STAT3 inhibition with the well tolerated and approved drug metformin may represent a promising new strategy for the treatment of high-grade glioma, but pharmacokinetic aspects, such as drug delivery to the brain and clinically achievable drug doses still need to be clarified.

## MATERIALS AND METHODS

### Ethics statement

The local department of neuropathology determined the patients’ diagnoses and WHO grade, and routine histopathology was accompanied by testing for IDH 1 mutation (by Sanger- or pyrosequencing) and MGMT promoter methylation status (by MethyQESD [70]). Clinical parameters such as age, gender, type of treatment, and overall survival (according to the RANO criteria) were available for all patients. The ethics committee of the University of Regensburg, Regensburg, Germany (No° 11-103-0182) approved the study and all patients gave written informed consent.

### Tumor cell lines

BTIC -7, -8, -10, -11, -12, -13, and -18 are primary tumor cell cultures derived from resected human glioblastoma as described before [19, 25]. For enrichment of BTICs, tumor specimens were mechanically (and partly also enzymatically) dissociated, washed with PBS and passed through a cell strainer with 30-μm pore size to obtain a single cell suspension (BD, #352235). Tumor cells were maintained in RHB-A based serum-free culture media (Takara, #Y40001), supplemented with 20 ng/ml of the mitogens EGF (#130097751) and bFGF (#130093842) (both Miltenyi Biotech), at 37 °C, 5% CO_2_, 95% humidity in a standard tissue culture incubator. Progenitor features of BTIC lines were verified by clonogenicity assays, and partly by tumor take assays in an immunocompromised mouse model. Differentiated TCs were generated via exposure of BTICs to 10% FBS (Biochrom, #S0115) in DMEM (#D6046) supplemented with 50 U (v/v) Penicillin, 0.05% (v/v) Streptomycin (#P4333), 2 mM (v/v) L-Glutamine (#G7513), 1% (v/v) MEM Vitamin Solution (#M6895) and 1% (v/v) non-essential amino acids (#M7145) (all Sigma-Aldrich) for at least 14 days.

### Proliferation assay

As described before [71], proliferation was assessed according to the manufacturers’ protocol by CyQUANT® Direct Cell Proliferation Assay (Thermo Scientific, #C35012). Briefly, cells were seeded considering their stereotypic growing characteristics in different amounts, i.e. at densities of 2.5, 3.75 and 5 × 10^4^ cells/ml, respectively, in 100 μl/well. Non-adherent cells were seeded on laminin- (Corning, #354 232) coated wells and incubated for 4 h. 72 h later the media was renewed and cell triplicates treated with specific concentrations of metformin, Stattic or a combination of both. 100 μl/well CyQuant Direct Solution were applied 1 h prior to measurement (excitation: 480 nm, emission: 535 nm). Proliferation was measured at the start of the assay (0 h), and at 48 and 96 h respectively. Blank values measured at every time point (100 μl media) and serial cell number dilutions served as references. For all assays, background fluorescence was subtracted and values were normalized to 0 h. Assays were performed in triplicates and repeated twice.

### Migration assay

Tumor spheroids were generated by seeding 5 × 10^3^ cells onto agarose-coated wells (1% agarose in 1x PBS) as described [19, 25, 71]. Cells were cultured for 48 h to allow spheroid formation. Mature spheroids were transferred into non-coated 96-well plates containing the corresponding drugs. Cell migration was monitored at 0, 16, 24, 40 and 48 h, taking into account the earliest time point when migration was measurable to prevent dilution of results by proliferation effects. The area covered by cells was measured manually (ImageJ software, NIH, USA) by an investigator. Assays were performed in triplicates and repeated twice.

### Organotypic brain slice cultures (OBSC)

OBSC were prepared according to Gogolla *et al*. [72] with customized modifications. Briefly, rat pups (postnatal day 12) obtained from an in-house facility (Long-Ewans, Sprague Dawley or Wistar) were killed by cervical dislocation and used to obtain OBSCs, as described [19].

48 h prior to implantation, lentivirally transduced BTICs were seeded onto agarose coated 96-well plates (10,000 cells/well) to allow for spheroid formation. BTIC-13, BTIC-12 and BTIC-10 were lentivirally transduced using a U57 pHR SFFV GFP plasmid while BTIC-8 was transduced with pLenti-H1-(shRNA-Neg-control)-Rsv(RFP-Bsd). One spheroid per hemisphere was placed onto the lateral ventricle, facing the hippocampal formation. Cell culture medium, also when containing different treatments, was changed every other day.

In order to monitor spheroid migration, implanted spheroids were visualized at 5-fold magnification under a fluorescent microscope (Zeiss Axio Observer.Z1, Visitron Systems GmbH, #3834003816) once a week. Several pictures of one infiltration site were merged when necessary using Pixelmator software, version 3.3.2.

### Protein isolation and western blot

To investigate protein levels of (p)STAT3, (p)mTOR, (p)AMPK, or GAPDH, whole-cell lysates were prepared with RIPA buffer (Perbio, #78440). For Western blot analysis, 30 μg of total cell lysates were diluted in Laemmli buffer, separated on a 10% SDS-PAGE gel and transferred to nitrocellulose membranes by semi-dry blotting. The membranes were blocked with 5% milk powder or 5% BSA in 0.02% Tween in TBS for 1 h. Membranes were incubated with specific monoclonal antibodies for STAT3 (#9145), pSTAT3 (phosphorylation site Y705, #9132 and S727, #9134), mTOR (#2983), pmTOR (phosphorylation site Ser2448, #5536), AMPK (#2603), pAMPK (phosphorylation site Thr172, #2535), all from Cell Signaling), or GAPDH (#sc-48167, Santa-Cruz) overnight at 4°C. Immunocomplexes were visualized using horseradish peroxidase-conjugated antibodies (goat anti-rabbit, Advansta #R-05072-500), donkey anti-goat (Santa-Cruz, #sc-2020)) followed by enhanced chemoluminescence (Western Bright Sirius ECL, Biozym #541-021). All Western blots were performed in duplicate. If several GAPDH controls are presented, the corresponding pairs are indicated by the same number of asterisks/pounds signs. Several GAPDH bands occurred, if we diluted not only 1x (30 μg) but 2x (60 μg) or more of the total cell lysates in Laemmli buffer and put the same sequence of cell lysates 2- times or more on the gel to reduce the number of stripping steps to evaluate several antibodies with similar molecular weight.

### RNA isolation and quantitative real time PCR (qRT-PCR)

For RNA isolation, cells were incubated in 6-well plates (2 x10^5^ cells per 2 ml). Total RNA was isolated by use of the Nucleo Spin RNA Plus Kit (Macherey-Nagel, #740 984.25) according to the manufacturer's instructions. Reverse transcription was performed with the Reverse Transcription System (Promega, #A3500) according to the manufacturer's protocol.

Quantification of STAT3 (forward: 5’-AAA GCA GCA AAG AAG GAG GC-3’, reverse: 5’-CTG GCC GAC AAT ACT TTC CG-3’) mRNA expression was performed by real-time PCR (Mx3000P Quantitative PCR [qPCR] System, Stratagene) based on SYBR-Green I fluorescence (Brilliant III Ultra Fast SYBR GREEN QPCR Master Mix, Agilent Technologies, #600883) using the ΔΔCT-method. RPLPO (large ribosomal protein) (forward: 5’-CTG TCT GCA GAT TGG CTA CCC-3’, reverse: 5’-GAT GGA TCA GCC AAG AAG GC-3’) served as housekeeping gene. Annealing temperatures were optimized for each primer pair. Three serial fivefold dilutions of cDNA, a mixture of all used cDNA-samples, were amplified in duplicates to construct standard curves for both the target gene and the reference (RPLPO, ribosomal protein, large, P0). cDNA-samples of BTICs were diluted 1:10. All samples were used in triplicates. For each reaction, melting curves were used to verify the identity of the amplification products. The target gene amount was divided by the reference (RPLPO) amount. Each of the experimental normalized values was divided by the normalized control (untreated) sample value to generate relative expression levels in fold changes

### Stable STAT3 overexpression

To achieve stable overexpression of STAT3, BTIC-8 and BTIC13 were lentivirally transduced with pLenti-Tet(CMV)-stat3C-Mut -Rsv(GFP-Puro). The plasmid was made according to the publication of Carro *et al*. [10] and purchased from AMS Biotechnology.

### Statistics

Analyses of significant differences between treatment groups (mean values and SDs) were performed by two-way ANOVA. We used Dunnett's test to control for multiple comparisons. The level of significance was set at *P<0.05, **P<0.01, ***<0.001 and ****P<0.0001. Data were analyzed using GraphPad Prism software (version 6, GraphPad Software, USA). In analyses comparing the combination of metformin and Stattic to the single agents, we used the asterisk to indicate significant differences as compared to the corresponding DMSO-control, whereas the pound sign indicates significance as compared to 1 mM metformin.

## SUPPLEMENTARY MATERIALS FIGURES AND TABLES


