# Supplementary material for: Stattic and metformin inhibit brain tumor initiating cells by reducing STAT3-phosphorylation
Source: Oncotarget. 2016 Dec 24;8(5):8250–63. doi: 10.18632/oncotarget.14159 (PMC5352398; doi:10.18632/oncotarget.14159)
Supplement: Supplementary file 1 [file oncotarget-08-8250-s001.pdf]

Stattic and metformin inhibit brain tumor initiating cells by reducing STAT3-phosphorylation

SUPPLEMENTARY FIGURES

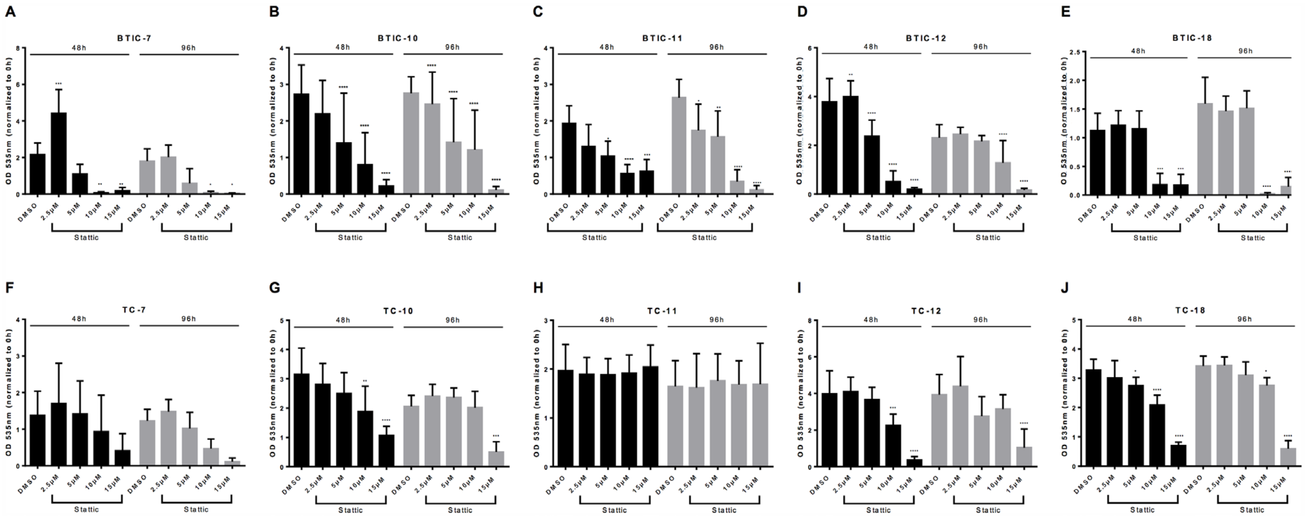

Supplementary Figure 1: Proliferation upon Stattic treatment for BTICs and corresponding TCs. A-E. Proliferation of BTIC-7, -10, -11, -12, -18 and **F-J.** Proliferation of TC-7, -10, -11, -12, and -18 as assessed by CyQuant Direct Cell Proliferation Assay®.

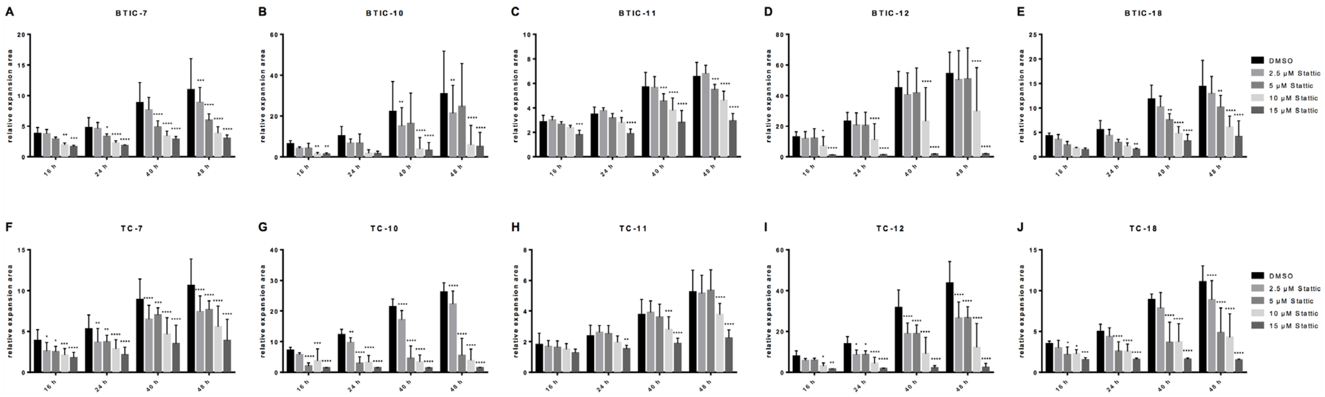

Supplementary Figure 2: Migration upon Stattic treatment for BTICs and corresponding TCs. A-E. Migration of BTIC-7, 10, -11, -12, -18 and **F-J.** TC-7, -10, -11, -12 and -18 in spheroid migration assays.

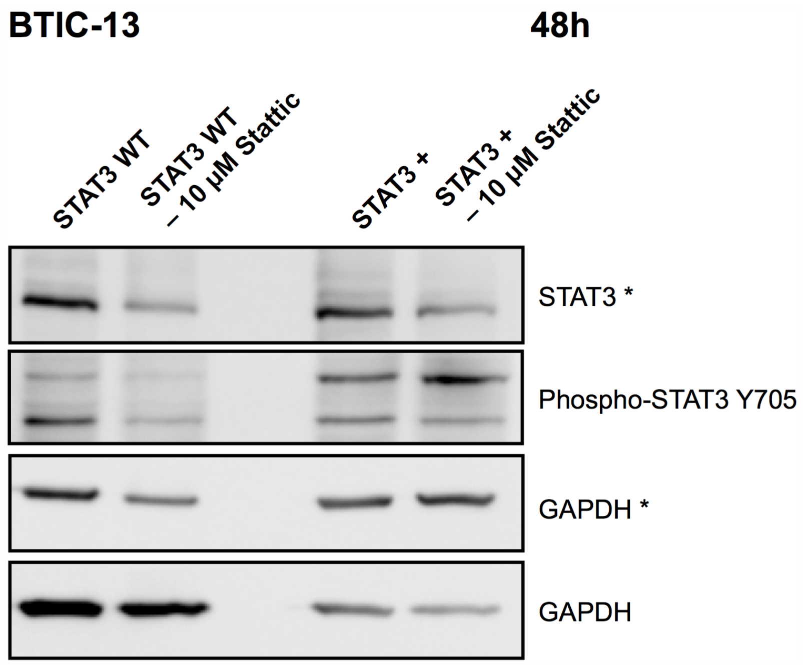

Supplementary Figure 3: Western blot analyses of wild type and STAT3-overexpressing BTIC-13 treated with or without 10 µM Stattic. Corresponding GAPDH controls are indicated by use or not use of the asterisk.

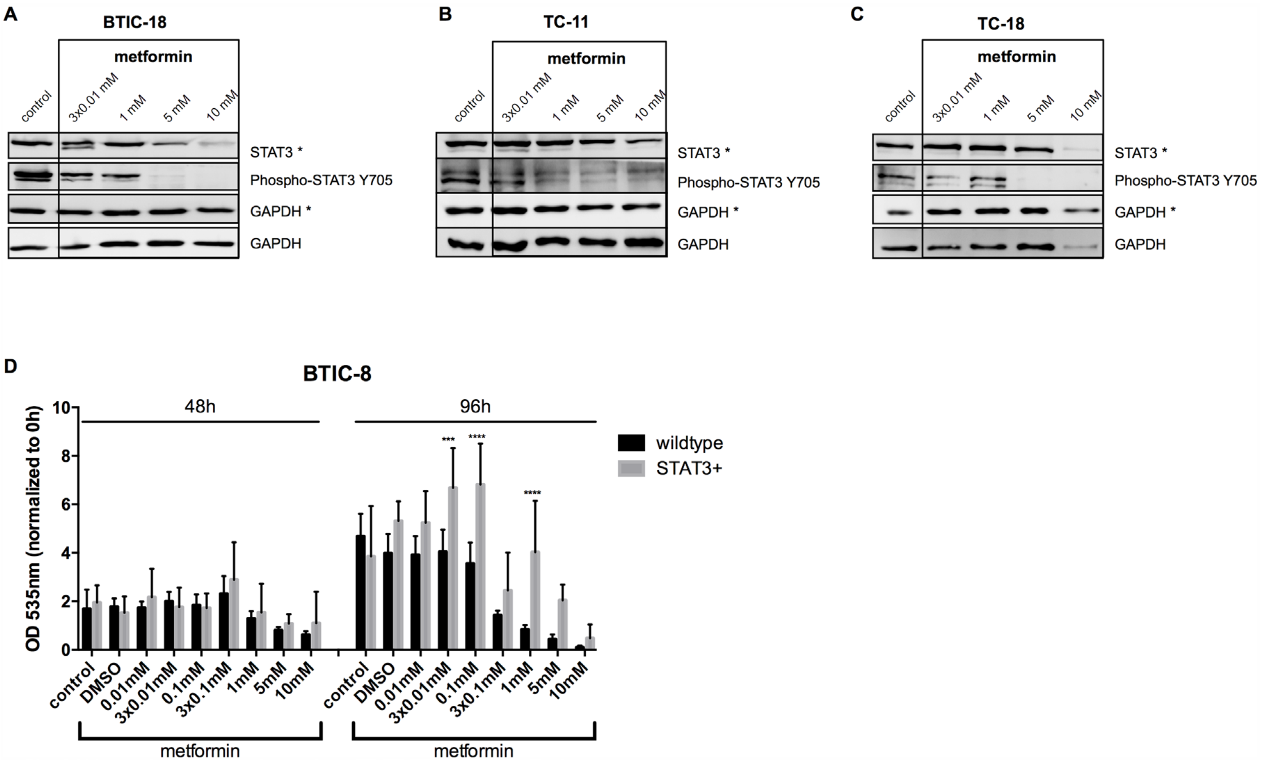

Supplementary Figure 4: Metformin inhibits phosphorylation of STAT3 in BTICs and TCs. Western blot analyses of BTIC-18 **A.**, TC-11 **B.** and TC-18 **C.** following metformin treatment revealed reduced STAT3-phosphorylation at Y705. Corresponding GAPDH controls are indicated by use or not use of the asterisk. Treatment of STAT-3 overexpressing BTIC-8 with metformin led to weaker inhibitory effects on cell proliferation **D.**

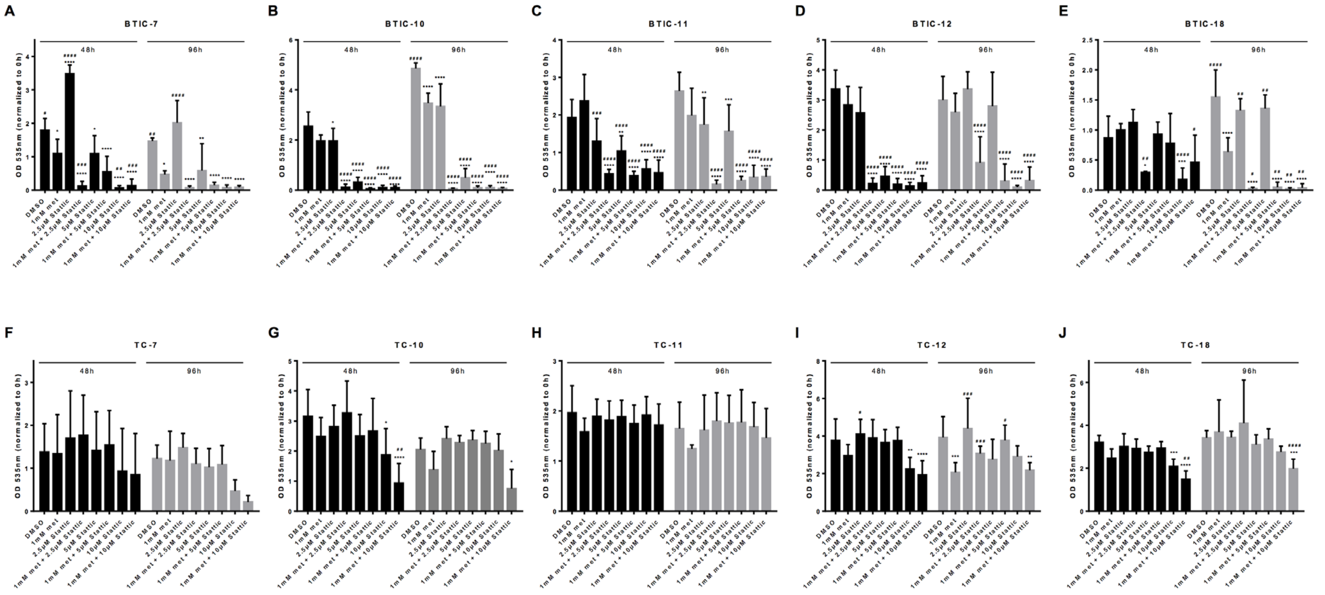

Supplementary Figure 5: Functional effects of the combination of metformin and Stattic on proliferation of BTICs. A-E. Proliferation of BTIC-7, -10, -11, -12, -18 and **F-J.** of TC-7, -10, -11, -12, and -18 following treatment with 1 mM metformin without or with the addition of 2.5, 5 and 10 µM Stattic, respectively. Asterisks indicate significant differences as compared to the corresponding DMSO-control, the pound signs indicate significance as compared to 1 mM metformin.

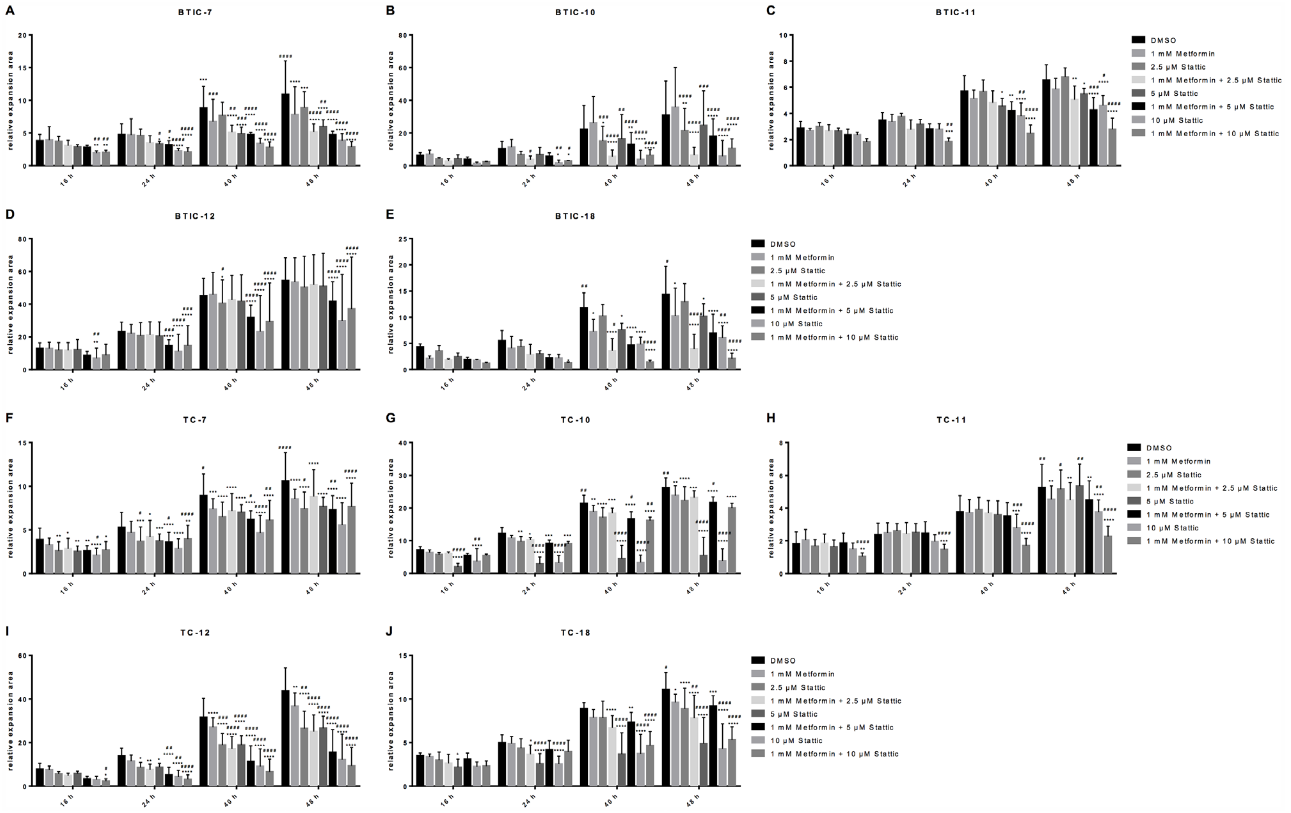

Supplementary Figure 6: Functional effects of the combination of metformin and Stattic on migration of BTICs. A-E. Migration of BTIC-7, -10, -11, -12, -18 and **F-J.** of TC-7, -10, -11, -12, and -18 after treatment with 1 mM metformin without or with the addition of 2.5, 5 and 10 µM Stattic, respectively. Asterisks indicate significant differences as compared to the corresponding DMSO-control, the pound signs indicate significance as compared to 1 mM metformin.

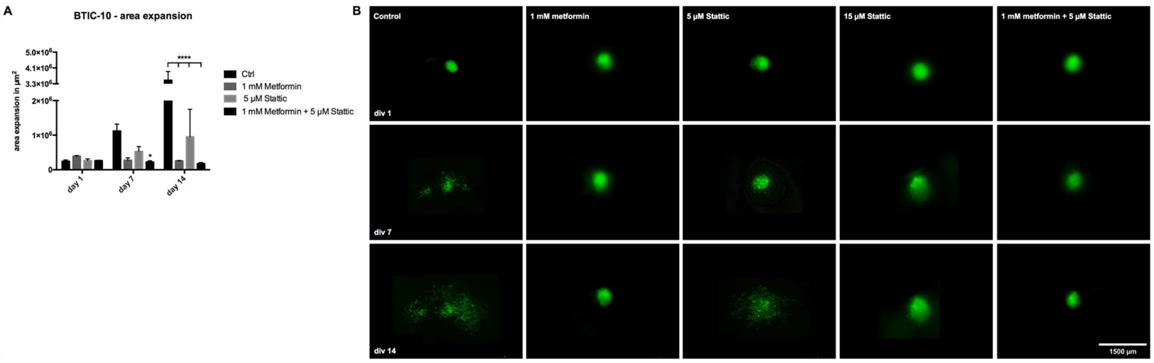

Supplementary Figure 7: Effects of STAT3 inhibition in BTIC-10 on OBSCs. A. Spheroid expansion area of BTIC-10 with or without treatment with 1 mM metformin, 5 µM Stattic and the combination thereof. Exemplary pictures of BTIC-10 are shown in **B.**
